# Improvement in Cerebral and Ocular Hemodynamics Early after Carotid Endarterectomy in Patients of Severe Carotid Artery Stenosis with or without Contralateral Carotid Occlusion

**DOI:** 10.1155/2016/2901028

**Published:** 2016-08-23

**Authors:** Jian Wang, Weici Wang, Bi Jin, Yanrong Zhang, Ping Xu, Feixiang Xiang, Yi Zheng, Juan Chen, Shi Sheng, Chenxi Ouyang, Yiqing Li

**Affiliations:** ^1^Department of Vascular Surgery, Union Hospital, Tongji Medical College, Huazhong University of Science and Technology, 1277 Jiefang Avenue, Wuhan 430022, China; ^2^Department of Ultrasound, Union Hospital, Tongji Medical College, Huazhong University of Science and Technology, 1277 Jiefang Avenue, Wuhan 430022, China; ^3^TCD Laboratory, Department of Neurology, Union Hospital, Tongji Medical College, Huazhong University of Science and Technology, 1277 Jiefang Avenue, Wuhan 430022, China; ^4^Department of Vascular Surgery, Fuwai Hospital, National Center for Cardiovascular Disease, Chinese Academy of Medical Sciences and Peking Union Medical College, 167 Belishi Road, Beijing 100037, China

## Abstract

*Purpose*. To investigate the alternation in cerebral and ocular blood flow velocity (BFV) in patients of carotid stenosis (CS) with or without contralateral carotid occlusion (CO) early after carotid endarterectomy (CEA).* Patients and Methods*. Nineteen patients underwent CEA for ≥50% CS. Fourteen patients had the unilateral CS, and five patients had the ipsilateral CS and the contralateral CO. Transcranial Doppler (TCD) and Color Doppler Imaging (CDI) were performed before and early after CEA.* Results*. In patients with unilateral CS, significant improvements in BFV were observed in anterior cerebral artery (ACA) and middle cerebral artery (MCA) on the ipsilateral side after CEA. In patients of ipsilateral CS and contralateral CO, significant improvements in BFV were observed in the ACA and MCA not only on the ipsilateral side but also on the contralateral side postoperatively. The ipsilateral ophthalmic artery (OA) retrograde flows in two patients were recovered to anterograde direction following CEA. The BFV in short posterior ciliary artery (SPCA) of the ipsilateral side significantly increased postoperatively irrespective of the presence of contralateral CO.* Conclusions*. CEA improved cerebral anterior circulation hemodynamics especially in patients of unilateral CS and contralateral CO, normalized the OA reverse flow, and increased the blood perfusion of SPCA.

## 1. Introduction

In patients with severe stenosis of internal carotid artery (ICA), carotid endarterectomy (CEA) has been shown to reduce embolic stroke risk [1, 2]. CEA certainly removes the atheromatous plaque in the carotid bifurcation, a possible source of cerebral emboli, and may prevent the progression of a stenosis to occlusion [3, 4]. Moreover, improvement of cerebral perfusion after CEA may further decrease stroke risk by a better washout of cerebral emboli from the border-zone areas [5].

Most reports have investigated the cerebral hemodynamic effect of CEA with interest being focused on the side of the operation in patients with unilateral carotid stenosis (CS) [6–8]. Contralateral carotid occlusion (CO) may be considered as a significant risk factor in CEA and results in the opening of cross flow through collateral pathways between two hemispheres. The hemodynamic changes in the hemisphere contralateral to the carotid stenosis (CS) early after CEA have been less studied [9], especially in patients of the severe CS with the contralateral CO [10–12]. The ophthalmic artery (OA) is the first branch of the ICA and can be an important collateral pathway between ICA and external carotid artery (ECA) in conditions of the severe CS and CO [13]. The reverse OA flow from ECA supplies the ipsilateral brain in response to reduced inflow pressure in the OA [14]. Previous studies reported that CEA resulted in significantly increased flow in the OA and that it corrected reversed flow in the OA in patients of severe CS [15–17]. Central retinal artery (CRA) and short posterior ciliary artery (SPCA) are two major terminal branches of OA, supplying all the structures in the orbit. To date, there are no available data on the changes of BFVs in the bilateral CRA and SPCA early after CEA, especially in patients of severe CS with contralateral CO.

The purpose of the study was to investigate alterations in cerebral and ocular blood flow in patients of the severe CS with or without the contralateral CO before and early after CEA. Additionally, the influence that ipsilateral CEA exerted on the occluded side was examined. Hemodynamic improvement was determined in the two hemispheres. Cerebral and ocular blood flow can be evaluated by transcranial Doppler (TCD) and Color Doppler Imaging (CDI), respectively. These simple noninvasive techniques provide information on blood flow velocities (BFVs) in cerebral and ocular artery vessels.

## 2. Methods

### 2.1. Subjects

Twenty-four consecutive patients with symptomatic ICA stenosis underwent CEA from November 2012 to October 2015 in our department. Five patients were excluded from this study because of the concurrent vertebral artery stenosis (≥30% diameter reduction). The remaining nineteen patients were finally enrolled in the study. Fourteen patients of the 19 (12 men and 2 women, age 66.2 ± 6.7 years) had unilateral CS (≥50%) with no or mild (<50%) stenosis on the contralateral side. The degree of the ipsilateral CS was 70%  ± 12.4%, ranging from 53% to 90%. Five patients of the 19 (3 men and 2 women, age 58.3 ± 9.3 years) had the ipsilateral CS (≥50%) and the contralateral CO. The degree of the ipsilateral CS was 76%  ± 15%, ranging from 60% to 91%. Eleven patients were operated on on the right side and eight on the left side. The hospital ethics committee approved the project, and all patients gave their informed consent. Clinical and angiographic manifestations of the patients were shown in Table 1.

The CS was assessed by duplex ultrasound and confirmed by CT angiography (CTA) of the supraaortic trunks. The degree of CS was calculated as the percentage of diameter reduction on the preoperative CTA. TCD and CDI examination before and 4.48 ± 2.59 days after CEA was a part of routine protocol accepted in our institution. CTA of carotid artery was also performed on the approximately 4th postoperative day to confirm the patent ICA. Neurologic complications during and after CEA were classified as transient ischemic attacks (TIA), minor disabling stroke or stroke lasting less than 7 days, and major stroke. We also registered symptoms of hyperperfusion syndrome (HS), such as headache, seizures, confusion, neurologic deficit, and high blood pressure (systolic blood pressure >150 mmHg/or diastolic blood pressure >90 mmHg). The intra- and perioperative cerebrovascular complications consisted of 2 patients with minor disabling stroke and no patients with HS.

### 2.2. Carotid Endarterectomy

All patients underwent surgery under general anesthesia more than one month after last ischemic attack. A longitudinal incision was made along anterior border of the sternocleidomastoid muscle to expose carotid sheath. Vascular clumps were used to occlude common carotid artery (CCA), ICA, and ECA. CCA and ICA were longitudinally opened along the anterior vessel walls. The atheromatous plaque and nearby intima were carefully removed from the carotid bifurcation. The arterial cutting was closed using patch and then vascular clamps were released. An intraluminal shunt was routinely used during the surgical procedure. The skin incision was eventually closed after ensuing vascular patency.

### 2.3. Transcranial Doppler

Transcranial Doppler sonography was performed by the same person to maintain a constant angle of insonation, with the patient lying in a comfortable supine position, with no visual or acoustic stimulation, in a quiet room. Recording was made using commercially available equipment (DWL Elektronische Systeme GmbH, Sipplingen, Germany) using a 2 Mhz pulsed Doppler probe. Anterior cerebral arteries (ACA), middle cerebral arteries (MCA), and posterior cerebral arteries (PCA) were insonated through the temporal window above the zygomatic arch at depths of 65–75, 50–60, and 60–75 mm, respectively. Basilar artery (BA) were insonated through the foramen magnum at a depth of 60–75 mm. BFV was expressed in cm/s as the peak value of the Doppler velocity spectrum outline (representing maximal flow velocity) over 4.5 s (*V*_peak_).

### 2.4. Color Doppler Imaging

The same experienced sonographer performed all retrobulbar CDI examinations by means of a color Doppler Imaging device (General Electric, Tokyo, Japan) using a 7.5 Hz multifrequency transducer. Patients were in the supine position with the upper body titled upward at about a 30-degree angle. Peak systolic velocity, defined as the BFV during the systolic phase of the cardiac cycle, and the end diastolic velocity, defined as the BFV at the end of the diastolic phase of the cardiac cycle, were measured in OA, CRA, and SPCA. The OA was identified as the vessel parallel to the nasal border of the optic nerve just after crossing it, the CRA as the vessel within the optic nerve and approximately 2–5 mm behind the globe, and the SPCA as the vessel on the temporal side of the optic nerve approximately 10–15 mm behind the globe.

### 2.5. Statistical Analysis

 Pre-CEA and post-CEA parameters were compared separately using two-tailed paired *t*-test. A* P* value of < 0.05 was considered statistically significant.

## 3. Results

### 3.1. The Effect of CEA on Cerebral and Ocular Blood Flow in Patients with Unilateral CS

Cerebral BFVs ipsilateral and contralateral to ICA stenosis in patients with unilateral CS after and before CEA were illustrated in Table 2. After CEA, the BFVs in the ipsilateral ACA and MCA increased from 108.02 ± 46.48 and 148.97 ± 77.06 to 126.91 ± 49.38 and 170.45 ± 83.82 cm/s, respectively. No significant differences in BFVs in the contralateral ACA and MCA were found between pre-CEA and post-CEA. Furthermore, no significant changes were seen in the BA and bilateral PCA after CEA.

Ocular BFVs ipsilateral and contralateral to ICA stenosis in patients with unilateral CS after and before CEA were illustrated in Table 3. In one patient undergoing CEA, retrograde flow in the ipsilateral OA was completely recovered to anterograde direction postoperatively. After CEA, the BFV in the ipsilateral SPCA increased from 9.39 ± 2.71 to 12.92 ± 4.01 cm/s. Nonetheless, no significant differences in BFVs in the contralateral SPCA and bilateral CRA were found between pre-CEA and post-CEA.

**Figure 1:**
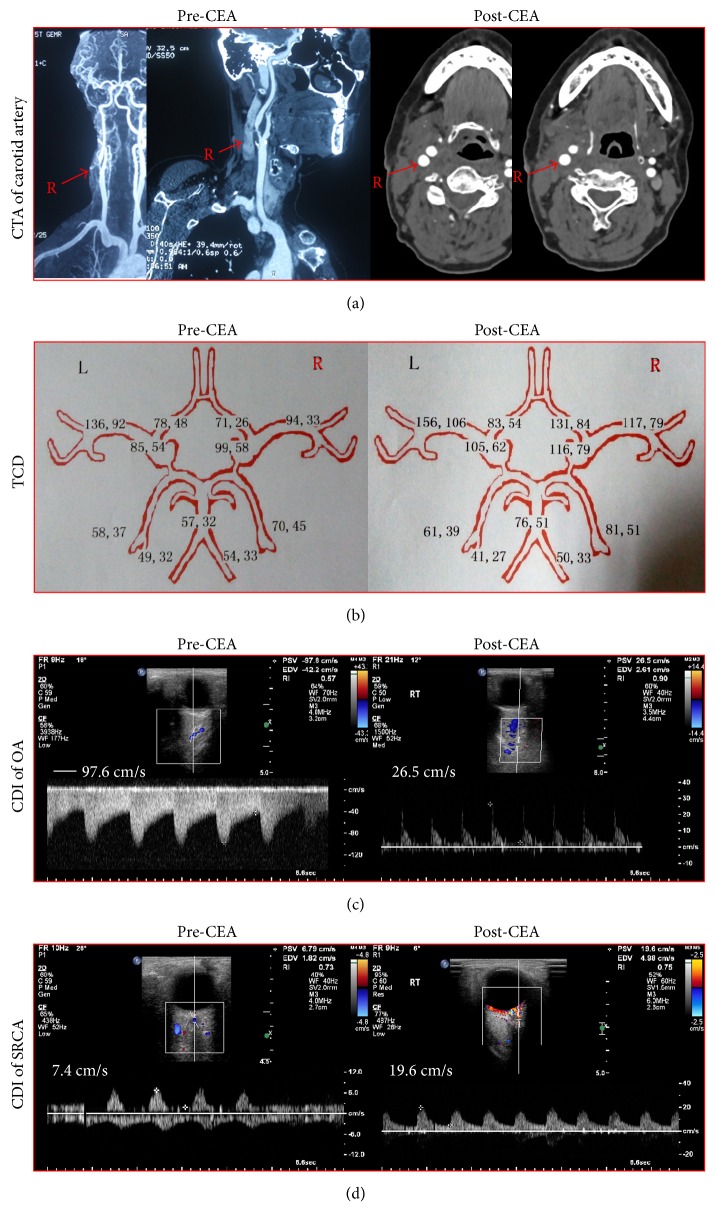
A 67-year-old woman complained of the amaurosis fugax and repeated transit ischemic attacks. (a) The patient had an 83% stenosis in the right ICA and experienced the uneventful right CEA. (b) CEA significantly improved the BFVs in the right ACA and MCA. (c) CEA normalized the reverse OA flow from right ECA. (d) CEA substantially improved the BFVs in the right SPCA.

Figure 1 showed the CTA of carotid artery, TCD, and ocular CDI of a 67-year-old woman before and after CEA. The patient had an 83% stenosis in the right ICA and underwent the successful right CEA. TCD showed that BFVs in the right ACA and MCA significantly increased postoperatively. CDI demonstrated the recovery of the reverse right OA flow and the markedly increased BFVs in the right SPCA after CEA.

### 3.2. The Effect of CEA on Cerebral and Ocular Blood Flow in Patients of the Severe CS with the Contralateral CO

Cerebral BFVs ipsilateral and contralateral to ICA stenosis in patients with severe CS and contralateral CO after and before CEA were shown in Table 4. After CEA, the BFVs in the ipsilateral ACA and MCA increased from 95.38 ± 17.17 and 119.01 ± 54.71 to 128.03 ± 29.88 and 154.12 ± 59.54 cm/s, respectively. Similarly, the BFVs in the contralateral ACA and MCA (95.47 ± 20.71 and 114.72 ± 78.33) also significantly increased postoperatively (131.46 ± 47.09 and 153.53 ± 85.06 cm/s). However, no significant changes were seen in the BA and bilateral PCA after CEA.

Ocular BFVs ipsilateral and contralateral to ICA stenosis in patients with severe CS and contralateral CO after and before CEA were shown in Table 5. In one patient undergoing CEA, retrograde OA flow in the ipsilateral side was completely changed to anterograde direction postoperatively. After CEA, the BFVs in the ipsilateral SPCA increased from 7.35 ± 1.36 to 12.4 ± 4.31 cm/s. Nonetheless, no significant differences in BFVs in the contralateral SPCA and bilateral CRA were found between pre-CEA and post-CEA.

**Figure 2:**
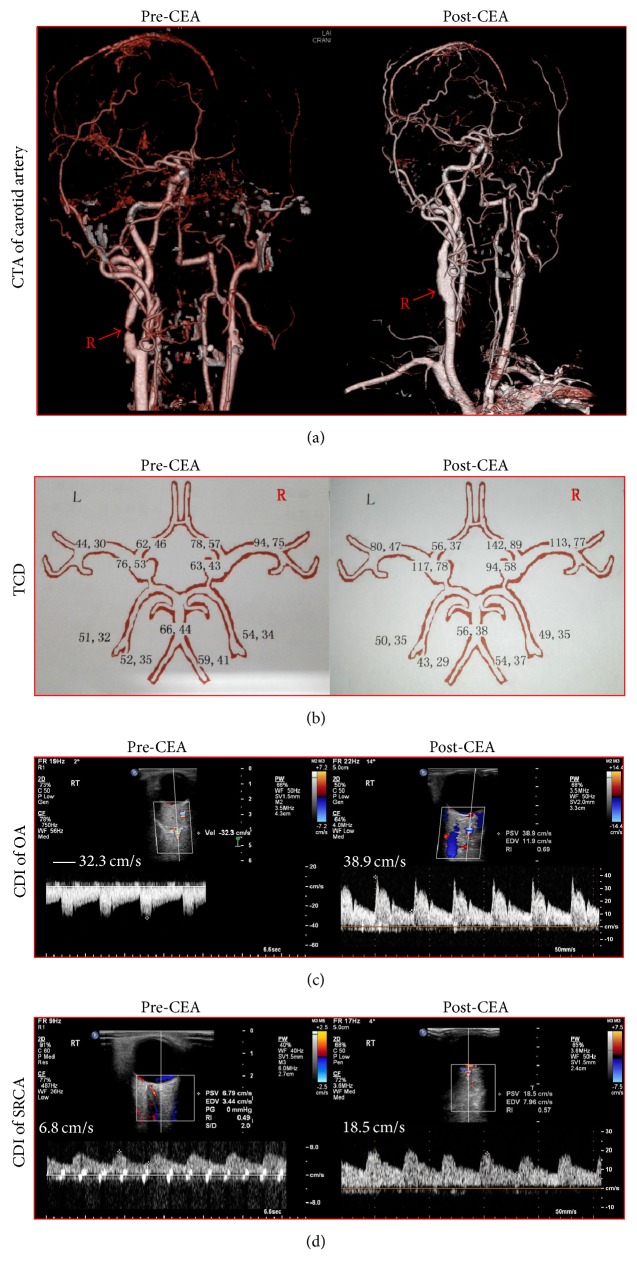
A 56-year-old man presented with vertigo, dizziness, and hemispheric stroke. (a) The patient had an 89% stenosis in the right ICA and the left ICA total occlusion and experienced the successful right CEA. (b) CEA significantly improved the BFVs in the right and left ACA and MCA. (c) CEA normalized the reverse OA flow from right ECA. (d) CEA substantially improved the BFVs in the right SPCA.

Figure 2 showed the CTA of carotid artery, TCD, and ocular CDI of a 56-year-old man before and after CEA. The patient had an 89% stenosis in the right ICA and a complete occlusion in the left ICA and underwent the successful right CEA. TCD showed that a significant improvement in cerebral BFVs of the ACA and MCA was observed not only in the right treated side but also in the left occluded side after CEA. CDI demonstrated the reversal of the retrograde flow in right OA and the markedly increased BFVs in the right SPCA postoperatively.

## 4. Discussion

Severe stenosis of the ICA resulted in a decreased arterial pressure distal to stenosis. Under normal circumstances, a decrease in regional cerebral perfusion pressure (CPP) is compensated for by a decrease in peripheral vascular resistance, by means of vasodilation (autoregulation) [18, 19]. As a result, the cerebral blood flow (CBF) can be maintained. Nonetheless, high-grade CS may be associated with the malfunction of vasodilation autoregulation and the exhaustion of the cerebral autoregulatory reserve capacity. Hino et al. reported that significant reduction in CBF was observed in the hemisphere not only ipsilateral but also contralateral to the stenosis in patients with severe ICA stenosis [20]. Van Laar et al. reported that regional CBF in the ipsilateral hemisphere (60.9 ± 16.9 mL/min/100 g) was significantly lower than in the contralateral hemisphere (70.9 ± 11.5) and control subjects (78.7 ± 18.4) in patients with unilateral high-grade ICA stenosis [8]. Meaningfully, CEA for unilateral CS resulted in a significant recovery increase in BFVs of ipsilateral ACA and MCA; and no significant changes were seen in the contralateral ICA, BA, and bilateral PCA in patients of severe unilateral CS. These changes can be interpreted as a consequence of the recovery of normal diameter, blood flow, and CPP in the ipsilateral ICA after CEA.

Similar results have been reported on the patients with unilateral severe CS after CEA. Jones et al. reported that ipsilateral supply to the MCA territory increased from 57.3 ± 5.7 to 67.3 ± 5.4 mL/100 g/min immediately after CEA and that a positive correlation was observed between obstruction ratio of ICA and change in supply to the ipsilateral MCA territory from the ipsilateral ICA [6]. van Laar et al. in 2006 reported that volume flow in the ipsilateral ICA increased from 114 ± 17 to 213 ± 17 mL/min, and no significant changes were seen in the contralateral ICA and BA one month after CEA [7]. They were also indicative of a positive correlation between the degree of stenosis and volume flow increase in the treated ICA [7]. van Laar et al. in 2007 demonstrated that regional CBF in the ipsilateral hemisphere increased from 60.9 ± 13.7 to 71.2 ± 13.9 mL/min/100 g one month after CEA [8]. Similarly, Sánchez-Arjona et al. found that MCA flow velocity on the ipsilateral side increased from 49.7 to 62.5 cm/s, and nonsignificant changes were seen on MCA of the contralateral side thirty days after carotid angioplasty stent placement (CAS) for ≥70% unilateral ICA stenosis [21].

When the patients suffered from the ipsilateral CS and the contralateral CO, differences in bilateral CPP promote the recruitment of collateral pathways. The ipsilateral ICA can compensate for decreased CPP via the circle of Willis in the contralateral CO. Primary collateral pathways are considered to be contralateral-to-ipsilateral cross flow via the anterior communicating artery and posterior-to-anterior flow via the posterior communicating artery; flow via the OA and leptomeningeal vessels are thought to be secondary collateral pathways recruited when collateral flow through the circle of Willis is inadequate. This study demonstrated a significant increase in bilateral ACA and MCA, and no significant changes were noted in the BA and bilateral PCA after CEA for the ipsilateral CS with the contralateral CO. The increased flow in anterior circulation not in posterior circulation suggests that collateral flow through the anterior circulation is important in the case of CEA-treated CS with the contralateral CO. CEA contralateral to CO increases the CPP in the ipsilateral hemisphere and enhances collateral flow via the anterior communicating artery to the hemisphere on the occluded side.

Our findings agreed with some previous studies on cases of severe CS with contralateral CO after CEA. Baracchini et al. found that CEA of the ipsilateral ICA stenosis improved CBF not only on the surgical side but also on the contralateral side of CO, and the proportion of patients with collateral flow via the anterior communicating artery increased significantly from 61.5% before to 89.7% within three months after CEA [10]. Kataoka et al. reported that the mean CBF of the treated side rose from 30.0 ± 7.1 to 34.4 ± 8.3 mL/min/100 g, and the mean CBF of the occluded side similarly rose from 28.3 ± 6.1 to 31.7 ± 6.4 mL/min/100 g eight to ten days after CEA [11]. They also indicated that there are significantly developed cross flow from the anterior communicating artery contributing to the improved CBF of the occluded side after CEA [11]. Rutgers et al. demonstrated that the BFV in the MCA of the occlusion side increased significantly from 71 to 85 mL/min at six months after CEA of the severe CS of the treated side, and the prevalence of collateral flow via the anterior communicating artery to the occlusion side increased significantly from 47% before to 84% after CEA [12].

In the study, a significant ocular hemodynamic improvement was observed after CEA, evidenced by the reversal of OA with retrograde flow and the increase in the BFVs of the ipsilateral SPCA. Ocular blood flow alteration occurred in the ipsilateral hemisphere in patients undergoing CEA for the severe CS irrespective of the contralateral CO. The hemodynamic effect of CEA, after removal of an atherosclerotic plaque, is an increase in CPP in the distal ICA. The OA is located downstream of the ICA, and the inflow artery for the OA is the ICA. Severe CS was associated with a marked reduction in OA and CRA flow velocities, which were corrected with successful CEA [15]. Kawaguchi et al. demonstrated that the BFV in OA increased from 9 ± 5 cm/s to 21 ± 5 cm/s one week after CEA [16]. Retrograde OA flow is suggestive of high-grade ICA stenosis and ipsilateral ECA collateralization of the OA with absence of a circle of Willis contribution. Rutgers et al. indicated that the proportion of reversed OA flow ipsilateral to severe CS decreased significantly from 42% before to 5% at six months after CEA [12]. Cohn Jr. et al. reported that eight patients with preoperative OA flow reversal had a return of normal OA flow within one month following CEA [15]. Zbornikova and Skoglund demonstrated that a change in the flow direction from being retrograde to antegrade was noted in 9/10 patients (90%) within forty-eight hours after CEA [17]. As a result, CEA improved chronic ocular ischemic syndrome associated with severe CS by increasing the OA blood flow and correcting the reversed OA flow [16].

Asymptomatic patients with substantial CS but no recent neurological symptoms are at increased long-term risk of the stroke, especially in the hemisphere ipsilateral to the CS. CEA has been shown to reduce the risk of ischemic stroke in patients with CS of ≥50% [22, 23]. A multicenter randomised trial involving 3120 patients demonstrated that stroke risks (immediate versus deferred CEA) were 4.1% versus 10.0% at 5-year follow-up and 10.8% versus 16.9% at 10-year follow-up [22]. The reduction in stroke risk following CEA has been correlated with the severity of CS ipsilaterally. Patients with severe CS of ≥70% had a dramatically reduced risk of ipsilateral stroke at eight years of follow-up [23]. CEA in patients with moderate CS of 50–69% yielded a moderate reduction in the risks of stroke [23]. Conversely, patients with stenosis of <50% did not benefit from the CEA [23]. The favorable benefit of CEA is attributable to the removal of the atheromatous plaque, which can be a source of cerebral emboli [3, 4]. Moreover, the improved CBF after CEA augmented the ability of the bloodstream to clear or wash out emboli and microemboli and restored available blood flow to regions rendered ischemic by emboli that block supply arteries [5].

The asymptomatic subjects with severe unilateral CS may be associated with an increased rate of cognitive impairment in the hemisphere ipsilateral to CS [24]. The presence of severe unilateral CS had an increased probability of developing cognitive deterioration especially in subjects with an associated hemodynamic impairment [25]. Furthermore, the subjects with asymptomatic bilateral severe CS were more likely to develop cognitive dysfunction compared to subjects with unilateral CS [26, 27]. The main mechanism linking CS and cognitive deterioration may be a result of chronic brain cortex hypoperfusion and impaired cerebrovascular autoregulation due to unfavorable hemodynamic changes [25–27]. CEA removes the atheromatous plaque, restores the CPP, improves the cerebral hemodynamics, and normalizes the cerebral metabolism. It was inferred that this cognitive impairment was improved with CEA [28, 29]. Heyer et al. indicated that CEA resulted in significantly increased CBF and improved cognitive performance as early as one day postoperatively [30]. Fearn et al. found that CEA restored the impaired cerebrovascular reserve and improved cognitive function at two months postoperatively [31]. Picchetto et al. demonstrated that the recanalization of a stenotic carotid improved brain cognitive function by resolving the chronic hypoperfusion at three months following CEA [32].

Our study has several limitations. First, the present study is the relatively small sample size. However, the sample size was enough to demonstrate significant improvement in cerebral and ocular hemodynamics after CEA. Second, cerebral and ocular hemodynamics were evaluated four days early after CEA. Further investigation needs to be carried out to clarify the long-term effect of CEA on cerebral and ocular BFVs.

## 5. Conclusions

In patients with unilateral CS undergoing CEA, ipsilateral hemodynamics of anterior circulation were significantly improved postoperatively. CEA contralateral to CO resulted in the significant improvement in hemodynamic of anterior circulation not only on the treated side but also on the occluded side. After CEA for the ipsilateral CS, collateral flow through anterior communicating artery compensated for the hemisphere on the occluded side in patients with the contralateral CO. CEA normalized the OA retrograde flow on the stenotic side and improved the blood flow in the ipsilateral SRCA irrespective of the contralateral CO.

## Figures and Tables

**Table 1:** Demographic data, risk factor, symptomatic characteristics, and degree of stenosis in the patients.

	Patients (*n* = 14) with the unilateral CS	Patients (*n* = 5) with the ipsilateral CS and contralateral CO
Age (years)	66.2 ± 6.7	58.3 ± 9.3
Sex (M/F)	12/2	3/2
Risk factors		
Smoking	10 (71.4%)	3 (60%)
Hypertension	9 (64.2%)	3 (60%)
Hyperlipidemia	7 (50%)	3 (60%)
Diabetes mellitus	4 (28.5%)	2 (40%)
CAD	4 (28.5%)	
POAD	4 (28.5)	
Symptom		
TIA including AF	9 (64.2%)	1 (20%)
Minor stroke	5 (35.7%)	3 (60%)
Major stroke		1 (20%)
Degree of CS		
50%–60%	4 (28.5%)	
60%–70%	3 (21.4%)	2 (40%)
70%–80%	4 (28.5%)	1 (20%)
80%–90%	3 (21.4%)	2 (40%)

CS, carotid stenosis; CO, carotid occlusion; CAD, coronary artery disease; POAD, peripheral obliterative atherosclerotic disease; TIA, transient ischemic attacks; AF, amaurosis fugax.

**Table 2:** Cerebral BFVs ipsilateral and contralateral to ICA stenosis in patients with unilateral CS after and before CEA.

	ACA (cm/s)	MCA (cm/s)	PCA (cm/s)	BA (cm/s)
Ipsilateral to ICA stenosis				
Pre-CEA	108.02 ± 46.48	148.97 ± 77.06	64.95 ± 17.73	75.05 ± 14.83
Post-CEA	126.91 ± 49.38^#^	170.45 ± 83.82^#^	68.63 ± 17.28	76.07 ± 16.63
*P* value	0.04406	0.02649	0.36967	0.77792
Contralateral to ICA stenosis				
Pre-CEA	121.24 ± 56.10	157.83 ± 57.29	64.61 ± 17.81	75.05 ± 14.83
Post-CEA	122.68 ± 32.08	163.81 ± 56.75	66.71 ± 21.27	76.07 ± 16.63
*P* value	0.89419	0.35183	0.68156	0.77792

BFV, blood flow velocity; ICA, internal carotid artery; CS, carotid stenosis; CEA, carotid endarterectomy; ACA, anterior cerebral artery; MCA, middle cerebral artery; PCA, posterior cerebral artery; BA, basilar artery.

^#^ *P* < 0.05 versus pre-CEA.

**Table 3:** Ocular BFV ipsilateral and contralateral to ICA stenosis in patients with unilateral CS after and before CEA.

	OA with retrograde flow (%)	CRA (cm/s)	SPCA (cm/s)
Ipsilateral to ICA stenosis			
Pre-CEA	1/13 (7.6%)	9.81 ± 2.95	9.39 ± 2.71
Post-CEA	0/13 (0%)	12.07 ± 5.10	12.92 ± 4.01^#^
*P* value	NA	0.07033	0.00996
Contralateral to ICA stenosis			
Pre-CEA	0/13 (0%)	10.67 ± 3.33	10.53 ± 3.75
Post-CEA	0/13 (0%)	12.13 ± 4.31	12.28 ± 4.21
*P* value	NA	0.25332	0.11588

BFV, blood flow velocity; ICA, internal carotid artery; CS, carotid stenosis; CEA, carotid endarterectomy; CRA, central retinal artery; SPCA, short posterior ciliary arteries; OA, ophthalmic artery; NA, not applicable.

^#^ *P* < 0.05 versus pre-CEA.

**Table 4:** Cerebral BFV ipsilateral and contralateral to ICA stenosis in patients with severe CS and contralateral CO after and before CEA.

	ACA (cm/s)	MCA (cm/s)	PCA (cm/s)	BA (cm/s)
Ipsilateral to ICA stenosis				
Pre-CEA	95.38 ± 17.17	119.01 ± 54.71	60.99 ± 12.45	94.61 ± 21.55
Post-CEA	128.03 ± 29.88^#^	154.12 ± 59.54^#^	70.21 ± 24.42	102.94 ± 33.86
*P* value	0.03489	0.00241	0.19174	0.50623
Contralateral to ICA stenosis				
Pre-CEA	95.47 ± 20.71	114.72 ± 78.33	67.02 ± 11.77	94.61 ± 21.55
Post-CEA	131.46 ± 47.09^#^	153.53 ± 85.06^#^	70.74 ± 18.99	102.94 ± 33.86
*P* value	0.04011	0.03536	0.35679	0.50623

BFV, blood flow velocity; ICA, internal carotid artery; CS, carotid stenosis; CO, carotid occlusion; CEA, carotid endarterectomy; ACA, anterior cerebral artery; MCA, middle cerebral artery; PCA, posterior cerebral artery; BA, basilar artery.

^#^ *P* < 0.05 versus pre-CEA.

**Table 5:** Ocular BFV ipsilateral and contralateral to ICA stenosis in patients with severe CS and contralateral CO after and before CEA.

	OA with retrograde flow (%)	CRA (cm/s)	SPCA (cm/s)
Ipsilateral to ICA stenosis			
Pre-CEA	1/5 (20%)	7.51 ± 2.02	7.35 ± 1.36
Post-CEA	0/5 (0%)	8.85 ± 2.25	12.4 ± 4.31^#^
*P* value	NA	0.38402	0.03723
Contralateral to ICA stenosis			
Pre-CEA	1/5 (20%)	6.67 ± 3.06	6.11 ± 1.29
Post-CEA	0/5 (0%)	7.66 ± 3.01	7.56 ± 1.97
*P* value	NA	0.08432	0.12419

BFV, blood flow velocity; ICA, internal carotid artery; CS, carotid stenosis; CO, carotid occlusion; CEA, carotid endarterectomy; CRA, central retinal artery; SPCA, short posterior ciliary arteries; OA, ophthalmic artery; NA, not applicable.

^#^ *P* < 0.05 versus pre-CEA.
